# Rapid Assessment of Mineral Concentration in Meadow Grasses by Near Infrared Reflectance Spectroscopy

**DOI:** 10.3390/s110504830

**Published:** 2011-05-03

**Authors:** Alastair Ward, Anne Lisbeth Nielsen, Henrik Møller

**Affiliations:** 1 Department of Biosystems Engineering, Aarhus University, Blichers Allé 20, DK-8830 Tjele, Denmark; E-Mail: henrikb.moller@agrsci.dk; 2 Natlan, Agro Business Park, Niels Pedersens Allé 2, DK-8830 Tjele, Denmark; E-Mail: lisbeth.nielsen@agropark.dk

**Keywords:** minerals, grassland, NIR, biogas, eutrophication

## Abstract

A near infrared reflectance spectroscopy (NIRS) method for rapid determination of nitrogen, phosphorous and potassium in diverse meadow grasses was developed with a view towards utilizing this material for biogas production and organic fertilizer. NIRS spectra between 12,000 cm^−1^ and 4,000 cm^−1^ were used. When validated on samples from different years to those used for the calibration set, the NIRS prediction of nitrogen was considered moderately useful with R^2^ = 0.77, ratio of standard error of prediction to reference data range (RER) of 9.32 and ratio of standard error of prediction to standard deviation of reference data (RPD) of 2.33. Prediction of potassium was less accurate, with R^2^ = 0.77, RER of 6.56 and RPD of 1.45, whilst prediction of phosphorous was not considered accurate enough to be of any practical use. This work is of interest from the point of view of both the removal of excess nutrients from formerly intensively farmed areas and also for assessing the plant biomass suitability for conversion into carbon neutral energy through biogas production.

## 1. Introduction

From 1951 to 2000 the area of permanent grassland outside rotation was reduced from 8.8 to 3.8% of the total area in Denmark [1]. In that period a substantial part of the riverside areas was included in intensive agricultural practices, which contributed to significant losses of N into the environment and subsequent eutrophication of water bodies [2]. An environmentally based increase in the percentage of extensively used grasslands mean that grass from these areas, as well as areas not under management at the moment, may be considered for use as a substrate in biogas plants for the production of renewable energy [3,4]. Besides energy production, anaerobic digestion of grass from meadows and other areas taken out of production will result in a transfer of nutrients to cash crop production which is of special interest in organic farming. Also the flora and fauna diversity of these areas are expected to benefit from harvest and removal of biomass from the sites.

However, there is little experience in using grass from such areas for biogas production and using the digestate as fertilizer in organic farming. To be able to assess the value of harvesting such areas with the aim to remove and transfer minerals to organic farming there is a need for a rapid method for assessing the mineral content. Furthermore, using a high proportion of grass for digestion might result in high ammonia content in the digester which can inhibit the process and lead to process failure [5]. A fast assessment of the nitrogen content in the raw material can give an early hint if the nitrogen load is likely to cause inhibition [6].

Typical methods of analysis for N, P and K are slow, labour intensive and may require a number of reagents, for example nitrogen determination by the Kjeldahl [7] or Dumas [8] methods. Near Infrared Reflectance Spectroscopy (NIRS) has proven to be a useful tool in the analysis of forages, as reviewed by Ren *et al.* [9]. NIRS provides a rapid and non-destructive analysis of several parameters simultaneously, although a calibration needs to be done first using the traditional measurement techniques as a reference. However, once an NIRS model has been made, measurements are quick and easy. When NIR radiation is absorbed by molecules the energy is converted to molecular vibration energy. Molecules which are infrared (IR) active are those which undergo a change in the dipole moment during transition, this means that bonds commonly found in biological systems such as C-H, O-H and N-H bonds are IR active. NIRS measurement of N, P, and K in soil has been previously demonstrated with good results for nitrogen whilst calibrations for phosphorous and potassium were less successful [10–12]. More specifically, when used for plant mineral determination, NIRS has been successfully used for measurement of nitrogen [13,14] and Vis-NIR (Visible and Near Infrared) for potassium [15], although less success has been achieved with the prediction of phosphorous [14,16].

In the present study, rapid assessment of the N, P, and K concentrations of meadow grasses by NIRS was investigated. The work was conducted on dry and ground forage samples from selected single plant species and mixed species grasslands at different harvest times over a four year period from several locations in central Jutland, Denmark.

## 2. Experimental Section

Samples were collected during the summer months of 2006–2009. Samples included single species as well as mixtures from diverse swards. The single species were *Deschampsia cespitosa* (L.) Be., *Juncus effusus* (L.), *Phalaris arundinacea* (L.) and *Holcus lanatus* (L.). The mixtures from diverse swards containing varying species and dominance of *Deschampsia cespitosa, Juncus effusus, Phalaris arundinacea, Holcus lanatus, Lolium perenne* (L.), *Festuca rubra* (L.), *Agrostis stolonifera* (L.), *Trifolium repens* (L.), *Glyceria fluitans* (L.) R. Br., *Glyceria maxima* (Hartman) Holmberg, *Poa pratensis* (L.), *Elytrigia repens* (L.) Nevski, *Taraxacum* sp. (L) and *Poa trivialis* (L.). Included in the swards were minor species, such as: *Alopecurus geniculatus* (L.), *Bromus mollis* (L.), *Agrostis tenuis* (Sibth.), *Agrostis gigantea* (Roth), *Achillea millefolium* (L.), *Cardamine pratensis* (L.), *Cerastium vulgare* (L.), *Cirsium palustre* (L.), *Epilobium palustre* (L.), *Hypochoeris radicata* (L.), *Ranunculus repens* (L.), *Ranunculus acris* (L.), *Rumex acetosa* (L.), *Rumex acetosella* (L.), *Rumex crispus* (L.), *Rumex obtusifolius* (L.), *Senecio jacobaea* (L.),*Trifolium pratense* (L.), *Urtica dioica* (L.), *Juncus articulates* (L.), *Juncus conglomeratus* (L.), *Juncus tenuis* (Wild.), *Luzula multiflora* (Retz.) Lej., *Equisetum arvense* (L.), *Equisetum palustre* (L.), *Carex leporina* (L.).

The samples were from sites with a big variation in productivity, from 3 to 9 t dry matter per ha per year, and most of the swards contained from 6–20 meadow species per 5 m^2^. The swards, from which the majority of the samples with different cutting times were collected, have been described earlier [17–20]. The total number of samples was 223 for nitrogen, 222 for phosphorous measurements and 183 for potassium. The potassium samples were all members of the larger N and P dataset.

Total nitrogen (TN) analysis was based on the Dumas method [8] using a Skalar Primacs SNC analyzer (Skalar Analytical B.V., Breda, The Netherlands). Total phosphorus (TP) and potassium were analyzed by Inductively Coupled Plasma (ICP) technology [21] (Perkin Elmer, Optima 2000DV, Waltham, MA, USA) after drying and destruction with H_2_NO_3_ in an autoclave.

A portion of each grass sample was dried at 60 °C for 48 h and ground to <0.8 mm using a Tekemas Christy mill 8-82-K (Chelmsford, UK). The dried and ground samples were subjected to NIRS analysis using a Bomen QFA Flex Fourier transform spectrometer (Q-Interline A/S, Roskilde, Denmark). The spectrometer was fitted with a powder sampler for rotating glass sample containers of 20 mL capacity (VWR / Bie & Berntsen A/S, Herlev, Denmark).

All multivariate analyses were carried out using The Unscrambler software (CAMO Software A/S, version 9.8, Oslo, Norway). The noise components of the NIR spectra were determined by visual examination and the region between 12,000 cm^−1^ and 4,000 cm^−1^ was used for subsequent analyses. The spectra were initially analyzed using principal component analysis (PCA). The NIR data was recorded as a matrix (*X*) of *p* columns representing absorbencies at different wavelengths and *n* rows representing objects or samples. PCA decomposes the *X* matrix of the NIR spectra into structural and noise components. The greatest variation in the data matrix is considered to be the first principal component (PC1) and the second greatest variation, PC2, is plotted orthogonally to PC1 and so on for further PCs. Higher order PCs progressively describe more noise and less structure in the *X* matrix. The models were validated using leave one out cross validation. The resulting PCA models can be visualized as a score plot which plots objects in relation to two PCs to show groupings and/or relationships.

The spectroscopic data was also used to create partial least squares (PLS) regression calibration models for determination of N, P, and K. All prediction models were created using the PLS-1 algorithm, suitable for single constituent determination and validated using test set validation. This validation method tests a calibration model by using it to predict values of a smaller sample set which was not used for the calibration. Variations between sampling years can cause problems for NIRS calibrations created using samples from one year when these are used to predict nitrogen content samples from a different year [22]. To test the robustness of the method, the large number of samples from the 2009 harvest year were used as a calibration set and the smaller number of samples from 2006–2008 used as a validation test set. The reference data used for calibration and validation are tabulated in Table 1.

The N validation set included some values higher than those used in calibration, which can be a good test of a calibration model [22]. The samples in the K validation set were not normally distributed, which is reflected in the relatively high mean value. However, the range of samples was similar to the calibration set. The single species samples were present in small numbers in both the calibration and validation datasets: *Phalaris arundinacea* (three calibration, five validation), *Juncus effuses* (two calibration, four validation), *Deschampsia cespitosa* (three calibration, five validation) and *Holcus lanatus* (two calibration, one validation).

The PLS regression models were optimized by mathematical data pre-treatments. Optimization of the N model was by unit vector normalization of the data. Normalization techniques attempt to scale the spectra. Unit vector normalization normalizes sample-wise data to unit vectors.

The optimum P model was found by modifying the spectral data by applying the Savitsky-Golay first order algorithm. This algorithm performs a least squares linear polynomial regression on each spectral point, the derivative is then calculated from the fitted polynomials at each point. In this case, the 1st order derivative was used and smoothed by two adjacent data points. Optimization of the K model required area normalization, which calculates and equalizes the area under each spectral curve.

Calibration models were evaluated based on four parameters: first, the coefficient of determination (R^2^), which indicates the proportion of variability explained by the model; second, the root mean square error of prediction (RMSEP), which is the average prediction error; Third, the ratio of Standard Error of Prediction (SEP) to the range of the reference values (RER); and finally, the residual prediction deviation (RPD), which is the standard deviation of the reference data divided by the RMSEP, thus relating the RMSEP to the range of the reference measurements. These parameters were used to classify the success of the predictions using the criteria described by Malley *et al.* [23] which are tabulated in Table 2. In addition to these criteria, some calibrations with R^2^ < 0.70 were considered to be useful for screening purposes [23].

## 3. Results and Discussion

### 3.1. Principal Component Analysis

Upon examination of the PCA score plot, no definite pattern was seen with the distribution of the samples for principal components one and two, which described 75% and 22% of the variance in the *X* matrix, respectively. However, there was some slight positioning of “June” samples to the upper left of the score plot. This pattern on the score plot with respect to harvest time was attributed to a change in the plant composition during growth.

Three samples were found to be standing apart from the group with respect to PC1 and were examined as being potential outliers. These samples were noted to have spectra with a reduced dynamic range compared to the other samples. All three of these samples are from the September 2009 harvest, but from three different fields with different species diversity. The biomass had been left to dry for a relatively long period at the field under non-optimal conditions. Still, this type of sample may occur and it led to the decision that these unusual samples were useful to the model so they were not discarded as outliers. Removing these samples from the analysis had little effect on the models, which gave further evidence that they were not spectral outliers.

Using PCA to look for patterns in the NIR spectra in relation to the sample diversity was not feasible due to the large number of sample types, and dividing the samples into different groups may cause problems in practical use.

### 3.2. Partial Least Squares Regression Analysis

**Figure 1.**
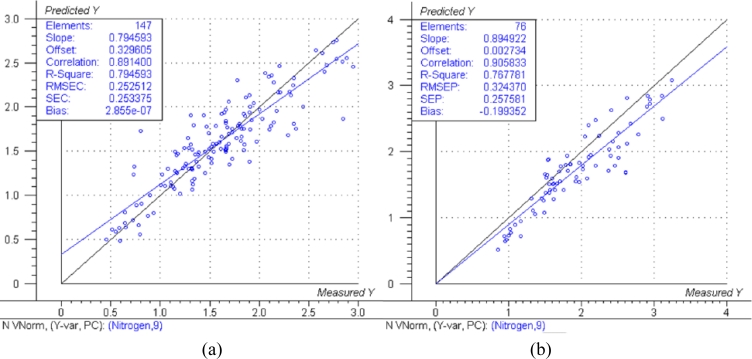
Predicted *vs.* reference plots and model data for: **(a)** Nitrogen calibration. (**b**) Nitrogen validation. The black lines represent the ideal 1:1 relationship between reference and predicted measurements, the blue lines represent the actual regression lines calculated in each calibration or validation.

**Figure 2.**
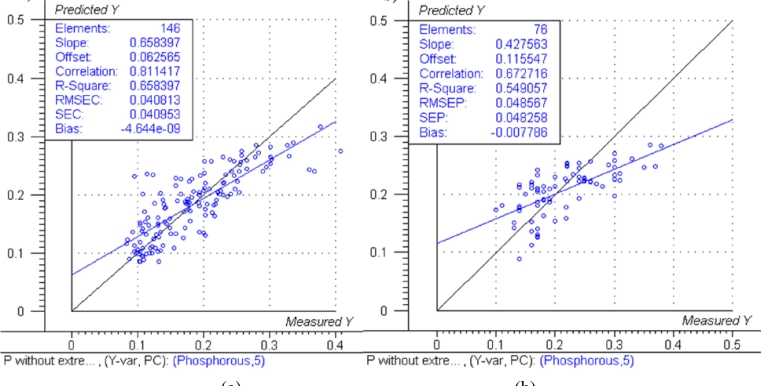
Predicted *vs.* reference plots and model data for: **(a)** Phosphorous calibration. (**b**) Phosphorous validation. The black lines represent the ideal 1:1 relationship between reference and predicted measurements, the blue lines represent the actual regression lines calculated in each calibration or validation.

**Figure 3.**
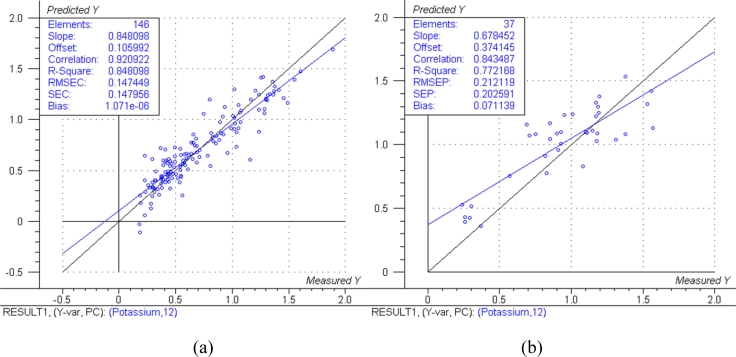
Predicted *vs.* reference plots and model data for: **(a)** Potassium calibration. (**b**) Potassium validation. The black lines represent the ideal 1:1 relationship between reference and predicted measurements, the blue lines represent the actual regression lines calculated in each calibration or validation.

The predicted *versus* measured plots with respective model performance data are shown in Figure 1(a), Figure 2(a) and Figure 3(a) for calibration models and Figure 1(b), Figure 2(b) and Figure 3(b) for test set validation predictions.

The RER and RPD values calculated from the validation SEP and reference data are summarised in Table 3.

Examining the performance data in Figures 1, 2 and 3 and those in Table 3, the nitrogen model can be classified as moderately useful, according to the criteria shown in Table 2 [23], although the RPD was high enough to be considered moderately successful.

The NIRS models for nitrogen prediction in grass presented here were less successful than those previously published. For determination of nitrogen in plant material by NIRS, Gislum *et al.* [13] reported R values of between 0.95 and 0.99 (R^2^ = 0.90 and R^2^ = 0.98 respectively) in *Festuca rubra* and *Lolium perenne* as single species, with RMSEP values as low as 0.19 % in a sample test set with a range of 0.80–5.92 %. Gislum *et al*. [13] suggested that a global model including all grass species and cultivars cannot be expected to have the same predictive ability as the two species model. The work presented here used samples from highly variable swards and as such can be considered to be more global than the Gislum study. However, excellent prediction of nitrogen by NIRS in botanically diverse grass samples with R^2^ values of at least 0.94 and RPD > 4 has been reported by Garcia-Ciudad *et al.* [22]. The reason for the considerable difference in calibration quality between the Garcia-Ciudad study and the results presented here is unclear. Both studies used botanically similar samples, dried and ground in similar ways. The Garcia-Ciudad [22] study used only six wavelengths in the 1,214–2,414 nm during their various calculations, some of which were based on previous experience with nitrogen and crude protein prediction by NIRS. Attempts to use the exact same wavelengths in this study produced models inferior to those presented in Figures 1,2 and 3 and Table 3, which were based on the full NIR range of 12,000 cm^−1^ to 4,000 cm^−1^ (833–2,500 nm). This suggests that errors in either the NIRS measurement or reference measurements were responsible for the poor model performance. The dried and ground material was well mixed before sub sampling for chemical and NIRS analysis. However, it was not possible in this study for both NIRS and reference analyses to be carried out on the exact same material, which could produce sampling errors [13]. Prediction of both phosphorous and potassium was less successful, falling below the criteria necessary to be considered moderately useful. The poor ability to predict phosphorous is in agreement with work carried out on soil [10] and *Phleum pratense* (Timothy grass) [16]. However, the prediction of potassium has been classified as successful in *Phleum pretense* in a study carried out by Tremblay *et al.* [16]. The prediction of potassium in this study can still be considered of use in making a simple estimation of high and low K concentrations.

Successful determinations of nitrogen by NIRS are clearly explained by the absorption of infrared radiation by the N-H bonds present in the plant dry matter, mainly in the proteins. However, P and K are not IR active. This can be attributed to K and to a lesser extent P being correlated to other, unknown compounds which are IR active [24]. The indirect measurement of P and K by NIRS could explain the reduced prediction accuracy of these models compared to the N prediction model. The meadow grass samples with different botanical composition used in this study had a much lower content of N, P and K than samples found in extensively managed, rotational clover grass at Danish organic farms, where the average of samples from 28 fields contained 2.7% N, 0.31% P and 2.3% K [25]. Including a wider range of samples to better represent Danish grassland would be likely to improve the robustness of the models.

The drying and grinding of the samples was performed for two reasons: first, the dry samples did not require immediate analysis and second it has been shown that such preparation provides better NIRS results [26] and have been used in many previous studies [13,16,22]. Ground samples are much more homogeneous than fresh samples and therefore more representative samples are presented to the NIR spectrometer. The use of dried and ground samples in this work could be considered a disadvantage due to the time required to prepare a new sample for analysis. However, the method including NIR-analyses was considerably faster and less labour intensive than the traditional methods as it allowed for quantification of N and approximate screening of K concentrations with one simple measurement and is open to future calibrations for other materials which may be of interest.

## 4. Conclusions

This work has shown that nitrogen in dried and ground meadow grass can successfully be quantified by NIRS. However, the prediction of potassium by NIRS was only suitable for simple screening of high and low values. Phosphorous prediction was not successful. These findings are of interest from both a nutrient removal and a bioenergy view point. Although only nitrogen prediction was classified as moderately useful, nitrogen is the main parameter of interest for biogas production, and an estimation of the mineral fertilizer value after treatment in a biogas plant is also of high interest, to optimize the subsequent use of fertilizer.

## Figures and Tables

**Table 1.** Reference data parameters. N, P and K data are shown as percentage of dry matter.

	
	**Parameter**	**N**	**P**	**K**

**Calibration**	*n*	147	146	146
	Min	0.46%	0.08%	0.18%
	Max	2.95%	0.41%	1.89%
	Mean	1.60%	0.18%	0.70%
	S. Dev	0.56%	0.07%	0.38%

**Validation**	*n*	76	76	37
	Min	0.85%	0.10%	0.24%
	Max	3.25%	0.38%	1.57%
	Mean	1.92%	0.22%	0.94%
	S. Dev	0.60%	0.07%	0.38%

**Table 2.** Calibration performance criteria according to Malley [23].

**Degree of calibration success**	**R^2^**	**RPD**	**RER**
Excellent	>0.95	>4	>20
Successful	0.90–0.95	3–4	15–20
Moderately successful	0.80–0.90	2.25–3	10–15
Moderately useful	0.70–0.80	1.75–2.25	8–10

**Table 3.** Calibration performance assessment data showing ratio of standard error of prediction to range of reference data (RER) and ratio of standard error of prediction to standard deviation of reference data (RPD).

**Parameter**	**N**	**P**	**K**
RER	9.32	5.80	6.56
RPD	2.33	1.45	1.88
